# Molecular Characteristic and Virulence Gene Profiles of Community-Associated *Methicillin-Resistant Staphylococcus aureus* Isolates from Pediatric Patients in Shanghai, China

**DOI:** 10.3389/fmicb.2016.01818

**Published:** 2016-11-15

**Authors:** Xing Wang, Xia Li, Wei Liu, Weichun Huang, Qihua Fu, Min Li

**Affiliations:** ^1^Department of Laboratory Medicine, Shanghai Children's Medical Center, Shanghai Jiaotong University School of MedicineShanghai, China; ^2^Department of Medical Microbiology and Immunology, University of California, DavisDavis, CA, USA; ^3^Department of Laboratory Medicine, Renji Hospital, Shanghai Jiaotong University School of MedicineShanghai, China

**Keywords:** community-associated methicillin-resistant *staphylococcus aureus*, MLST-genotyping, SCCmec typing, spa typing, virulence factors

## Abstract

*Staphylococcus aureus* is a globally important human pathogen, especially among children and immunocompromised patients. The emergence and spread of community-associated methicillin-resistant *S. aureus* (CA-MRSA) has become a serious public health problem worldwide. The aim of this study was to investigate the prevalence, molecular characteristics and virulence profiles of CA-MRSA infections from pediatric patients in a university hospital in Shanghai, China. A total of 80 CA-MRSA isolates were collected from July 2012 to December 2013 in Shanghai Children's Medical Center and analyzed by multilocus sequence typing, staphylococcus chromosomal cassette *mec* (SCC*mec*) typing, and *spa* typing. The detection of Panton-Valentine Leukocidin (*pvl*), superantigenic and exfoliative toxins, and adhesin genes was also performed. Overall, 16 distinct sequence types (STs) were identified among the 80 isolates. Among them, ST59 was found to be the most prevalent, followed by ST398 (11.3%, 9/80) and ST88 (8.8%, 7/80). SCC*mec* types IV and V were observed, at 60 and 40%, respectively. Thirty *spa* types were identified, *spa t*437 (23.8%) was the most predominant type. All 80 isolates exhibited carriage of at least four virulence genes. Thirty-four (42.5%, 34/80) isolates harbored ≥10 tested virulence genes. Adhesion genes were present in most of the MRSA isolates, including the following: *icaA* (100%), *clfA* (100%), *sdrC* (95%), and *sdrE* (63.8%). The prevalence of *pvl* gene was 20%, and multidrug resistance was observed in 36% of all strains. In addition, ST59-MRSA-IV with *t*437 accounted for 21.3% of occurrences, making it the most prevalent clone. Isolates that were carriers of toxin genes, and *hla* (100%) and *hlg* (87.5%) were the most frequent. In conclusion, simultaneous carriage of multiple virulence genes and genetically considerable diversity were very common among CA-MRSA from pediatric patients in Shanghai. ST59-MRSA-IV with *t*437 was still the most predominant type. The combination of virulence gene profiles and antibiotic resistance may help ST59 to be successfully spread among children.

## Introduction

*Staphylococcus aureus* is one of the most prevalent human pathogens, causing a broad variety of diseases ranging from mild skin and soft-tissue infections to severe systemic infections such as sepsis and necrotizing pneumonia (Lowy, 1998; Deresinski, 2005). Since methicillin-resistant *S. aureus* (MRSA) was first reported in the United Kingdom in 1961 (Monecke et al., 2011), it has become a particular public threat to human health. During the last 55 years, various hospital-associated MRSA (HA-MRSA) clones have disseminated worldwide, including Europe, United States, North Africa, the Middle East, and East Asia. Since the 1990s, community-associated MRSA (CA-MRSA) emerged as a serious health problem worldwide (David and Daum, 2010; DeLeo et al., 2010), first in communities and later in healthcare facilities. Compared to HA-MRSA strains, most CA-MRSA isolates that harbor SCC*mec* types IV or V, do not have multi-antibiotic resistance (except to β lactams), and many possess different exotoxin gene profiles (e.g., PVL genes; Dinges et al., 2000). However, another MRSA clone from animals (LA-MRSA) has emerged in humans exposed to livestock since 2005. Livestock-associated MRSA ST398, isolated from pigs and pig farmers has been reported in European countries and North America. For LA-MRSA, ST398 is the overwhelmingly dominant lineage in Europe, whereas ST9 predominates in most Asian countries (Fluit, 2012; Chuang and Huang, 2015).

Currently, more than 20 genetic lineages have been reported to be associated with CA-MRSA worldwide (Witte, 2009; Mediavilla et al., 2012). The top five major lineages include ST1-IV (WA-1, USA400), ST8-IV (USA300), ST30-IV (South West Pacific clone), ST59-V (Taiwan clone), and ST80-IV (European clone). However, CA-MRSA strains were found to be continent-specific. For example, ST1 and ST8 clones are mostly found in the United States and Canada, whereas ST80 clones are mainly found in Europe. In China and several other Asian countries, ST59-IV and ST59-V are the most common CA-MRSA strains. The prevalence of CA-MRSA varies greatly and the occurrence rate of CA-MRSA infections ranges from <5 to >35% in different Asian countries (Chen and Huang, 2014). Prevalence is higher in children than in adults. Because of low immunity in children, once infected with CA-MRSA, the consequences are often very serious (Machuca et al., 2013). Although, CA-MRSA infection and transmission represent a major public health problem, there is limited information reported on CA-MRSA monitoring among children in China. The aim of this study was to investigate the prevalence, molecular characteristics and virulence profiles of CA-MRSA infections from pediatric patients in China and to determine whether the epidemiology could be affected by time.

## Materials and methods

### Bacterial isolates

From July 2012 to December 2013, a total of 80 sequential CA-MRSA isolates, which represent all the non-duplicate strains isolated during the study period, were collected from pediatric patients with local or systemic infections in a university hospital in Shanghai, China (Shanghai Children's Medical Center, affiliated with Shanghai Jiao Tong University; CLSI, 2012). Shanghai Children's Medical Center is one of the largest pediatric hospitals in China with 800 beds and ~3000 hospital admissions per day. MRSA isolates were confirmed by classic microbiological methods: Gram stain and catalase and coagulase activity on rabbit plasma. They were further identified by biochemical characterization using the Api-Staph test (bioMérieux, Lyon, France).

CA-MRSA was defined as an MRSA isolate that was obtained either from an outpatient or an inpatient within 48 h of hospitalization, and without the patient having medical history of MRSA infection or colonization, admission to a healthcare facility, dialysis, surgery or insertion of indwelling devices in the past year.

These isolates were recovered from several clinical sources, including the respiratory tract (sputum, pharynx swabs, and bronchial alveolar lavage fluid), skin and soft tissue (cutaneous abscess and wound secretion), cerebrospinal fluid, blood and urine (Klevens et al., 2007). All strains were stored at −70°C and grown overnight on sheep blood agar plates at 37°C.

The Ethics Committee of Shanghai Children's Medical Center exempted this study from review because the present study focused on bacteria.

### Antimicrobial susceptibility testing

The antibiotic susceptibility of all isolates in this study was performed using the bioMe'rieux VITEK2 system following manufacturer's instructions. Results were interpreted in accordance with Clinical and Laboratory Standards Institute (CLSI) guidelines (CLSI, 2012). The following 17 drugs were tested: cefazolin (CFZ), linezolid (LZD), ciprofloxacin (CIP), clindamycin (DA), erythromycin (E), trimethoprim-sulfamethoxazole (SXT), moxifloxacin (MOF), nitrofurantoin (FD), vancomycin (V), tetracycline (TET), penicillin (P), rifampicin (RF), levofloxacin (LVX), ampicillin (AMP), gentamicin (GM), quinupristin/dalfopristin (Q/D), and tigecycline (TGC). *S. aureus* ATCC 29213 was used as a quality control.

### MLST analysis

Isolates were screened according to the protocol described (Enright and Spratt, 1999) on the *S. aureus* MLST website (http://saureus.mlst.net) to detect the following seven housekeeping genes (Aanensen and Spratt, 2005): carbamate kinase (*arcC*), shikimate dehydrogenase (*aroE*), glycerol kinase (*glp*), guanylate kinase (*gmk*), phosphate acetyltransferase (*pta*), triosephosphate isomerase (*tpi*), and acetyl coenzyme A acetyltransferase (*yqiL*). PCR amplicons of seven *S. aureus* housekeeping genes were obtained from chromosomal DNA. The sequences of the PCR products were compared with the existing alleles available from the MLST website, and the allelic number (sequence type, ST) was determined for each sequence. Clustering of related STs, which were defined as clonal complexes (CCs), was determined using eBURST (based on related STs).

### SCC*mec* typing

The MRSA isolates were subjected to SCC*mec* typing as described by Kondo et al. (2007), which was based on a set of multiplex PCRs (M-PCRs) with 14 primers. SCC*mec* types I–V were assigned according to the combination of the cassette chromosome recombinase (*ccr*) type and *mec* class. MRSA isolates that could not be assigned to any expected type were defined as nontypable (NT).

### *Spa* typing

In *S. aureus*, the polymorphic X region of staphylococcal protein A (*spa*) gene consists of a variable number of 24 bp repeat units (Shopsin et al., 1999) that allow isolates to be distinguished from one another. The *spa* typing was based on variations of the repeat units. Amplification and sequencing of the X region were performed as described previously by Koreen et al. (2004). The *spa* typing was assigned by submitting the data to the *S. aureus spa* type database (http://spaserver.ridom.de).

### Virulence gene profiles

All MRSA isolates were screened for the following 22 staphylococcal virulence genes: the staphylococcal enterotoxin genes (*sea, seb, sec, sed, see, seg, seh, sei, seq, sek*), the toxic shock syndrome toxin (*tsst*), the arginine catabolic mobile gene (*arcA*), the exfoliative toxin genes (*eta, etb*), the PVL genes (*luk*F/S-PV; Lina et al., 1999), the hemolysin gene (*hla, hlb, hlg*), and the adhesin genes (*clfA, icaA, sdrC*, and *sdrE*) as previously described (Arvidson and Tegmark, 2001; Peacock et al., 2002; Bubeck Wardenburg et al., 2007).

### Statistical analysis

Statistical analyses were performed using Stata software (version 10.1/SE, Stata Corp., College Station, TX, USA). We used the χ^2^ and Fisher's exact tests, as appropriate for analysis of categorical data. Statistical significance was set at *P* ≤ 0.05.

## Results

### Clinical features

Eighty MRSA isolates were obtained from patients 10 days to 11 years old with local or systemic infection and all the isolates were positive for the *mecA* genes. Among them, 52 patients (65%) were male. 61.3% (49/80) of the children were <1 year old and 18 were neonates (22.5%). From the clinical medical records, respiratory infection was the most frequently determined infection type caused by CA-MRSA; 82.5% (66/80) of the isolates were from the respiratory tract, and 5% (4/80) of the isolates were associated with bloodstream infection (BSI).

### MLST, SCC*mec*, and *spa* typing

**Figure 1:**
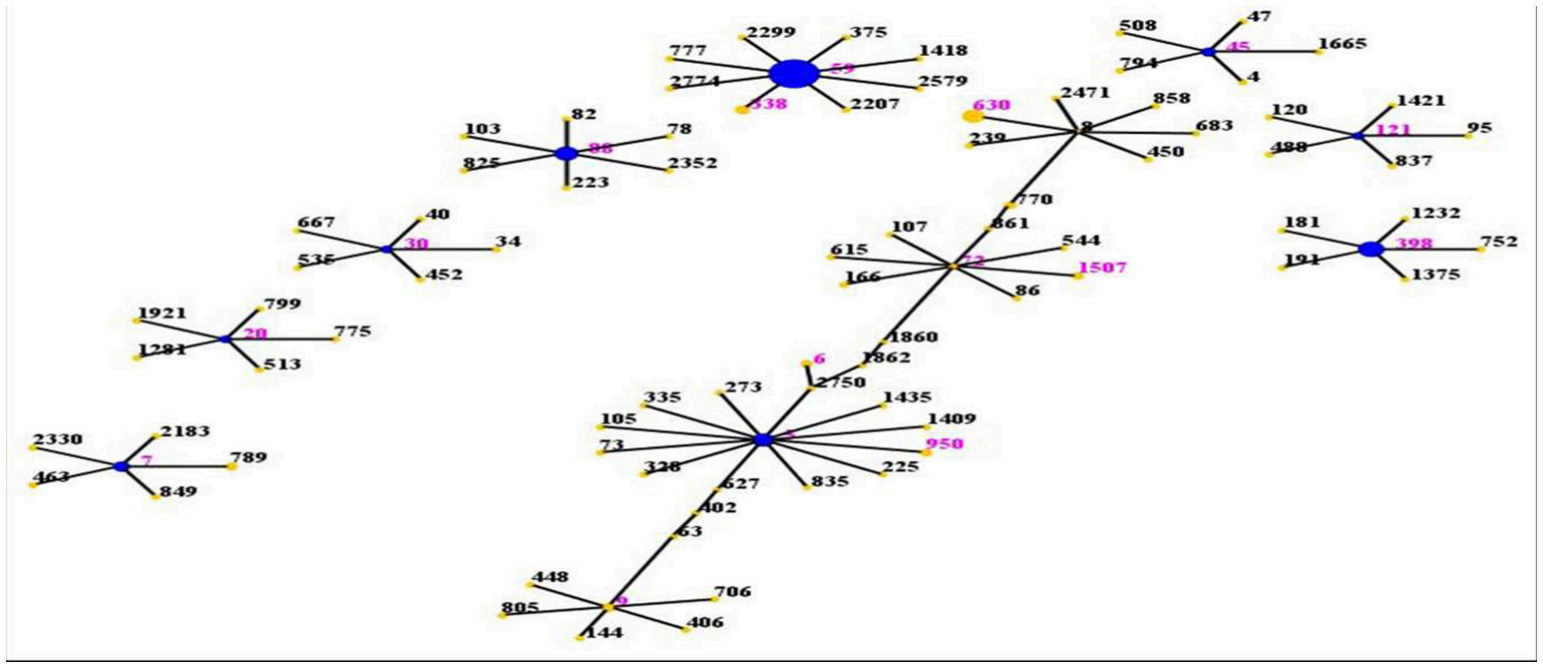
**Distribution of STs in the clonal complexes**. The eBURST application of the MLST data from all of the isolates analyzed in this study. The purple numbers represent 16 STs which are found in 80 MRSA isolates. STs that are linked by a line belong to the same cluster. Circle sizes are proportional to the number of strains within the ST.

The evolutionary and genetic diversity of MRSA isolates within individual patients was analyzed by MLST (Table 1). There were 16 distinct STs identified within the 80 isolates, among which the most frequently represented was ST59 (45.0%, 36/80). It accounted for nearly one half of all MRSA isolates, followed by ST398 (11.3%, 9/80), ST88 (8.8%, 7/80), ST5 (7.5%, 6/80), ST630 (7.5%, 6/80), ST7 (3.8%, 3/80), ST338 (2.5%, 2/80), ST45 (2.5%, 2/80), ST9 (2.5%, 2/80). Among the remaining 7 STs (ST30, ST72, ST6, ST20, ST950, ST1507, ST121), each ST just had one isolate. This methodology revealed that the strains clustered by eBURST into 9 CCs (CC59, CC398, CC88, CC5, CC8, CC7, CC72, CC45, CC9) and 4 singletons (Figure 1), among which were CC59 with 38 isolates, CC398 with 9 isolates, CC88 with 7 isolates, CC5 with 7 isolates, CC8 with 6 isolates, CC7 with 3 isolates, CC72 with 2 isolates, CC45 with 2 isolates, and CC9 with 2 isolates. Sixteen isolates harboring *pvl* were distributed among 7 different STs, including ST59 (*n* = 9) and ST338 (*n* = 2), as well as ST398, ST88, ST30, ST20, and ST121 (one isolate each).

**Table 1:** **Clonal complexes and the relationship among the molecular types of MRSA isolates from pediatric patients**.

**Clonal complex (CC)**	**MLST (*n*,%)**		**SCCmec type**		
		***spa* Type**	**IV**	**V**	**NO**.
CC59	ST59 (36, 45.0%)	*t*437	14	3	17
		*t*441		7	7
		*t*316	4		4
		*t*172	4		4
		*t*163	1		1
		*t*1751	1		1
		*t*3523	1		1
		*t*8886	1		1
	ST338 (2, 2.5%)	*t*437		2	2
CC398	ST398 (9, 11.25%)	*t*034	2	4	6
		*t*011	1	1	2
		*t*1250		1	1
CC88	ST88 (7, 8.75%)	*t*2310	4		4
		*t*7480	2		2
		*t*7637		1	1
CC5	ST5 (6, 7.5%)	*t*062	3		3
		*t*2460	1		1
		*t*187		1	1
		*t*002	1		1
	ST950	*t*895	1		1
CC8	ST630 (6, 7.5%)	*t*4549	1	4	5
		NT		1	1
CC7	ST7 (3, 3.75%)	*t*796		2	2
		*t*091	1		1
CC45	ST45 (2, 2.5%)	*t*116	1		1
		*t*230	1		1
CC72	ST72	*t*664	1		1
	ST1507	*t*664	1		1
CC9	ST9 (2, 2.5%)	*t*899		2	2
	ST30	*t*019	1		1
	ST6	*t*701		1	1
	ST20	*t*164		1	1
	ST121	*t*159		1	1

By SCC*mec* typing, only two types (types IV and V) were found among 80 MRSA isolates. The most common was type IV, which was found in 48 isolates (60%, 48/80), while type V was found in 32 isolates (40%, 32/80).

The *spa* typing discriminated MRSA isolates into 30 *spa* types. Among them, *spa t*437 was the most predominant type (23.8%, 19/80), followed by *t*441 (8.8%, 7/80), *t*034 (7.5%, 6/80), *t*4549 (6.3%, 5/80), *t*172 (5%, 4/80), *t*316 (5%, 4/80), and *t*2310 (5%, 4/80). Each of the remaining *spa* types was represented in <3 isolates.

There was a strong association observed between specific ST and *spa* types. The ST59 genotype was associated primarily with *spa t*437 (47.2%, 17/36) and *spa t*441 (19.4%, 7/36), and less frequently with six types: *t*316, *t*172, *t*163, *t*3523, *t*8886, *t*1751. The ST398 genotype was associated mainly with *spa t*034 (6/9) and *spa t*011 (2/9).

### Antimicrobial susceptibility testing

The antimicrobial resistance profiles of 80 MRSA isolates according to MLST are listed in Table 2. All the strains were resistant to cefazolin, penicillin, and ampicillin, but susceptible to vancomycin, linezolid, nitrofurantoin, quinupristin/dalfopristin, and tigecycline. The majority were resistant to clindamycin (80%) and erythromycin (82.5%), however, they were susceptible to most of the antibiotics tested. The resistance rates to other antibiotics tested were 8.8% to ciprofloxacin, 10% to trimethoprim-sulfamethoxazole, 3.8% to moxifloxacin, 32.5% to tetracycline, 2.5% to rifampicin, 5% to levofloxacin, and 3.8% to gentamicin. The resistance profiles of MRSA isolates differed by their STs. For the ST59 strains, the most prevalent antibiotic group contains clindamycin, erythromycin, and tetracycline, while ST9 strains were more resistant to more antibiotics including ciprofloxacin, clindamycin, erythromycin, trimethoprim-sulfamethoxazole and tetracycline.

**Table 2:** **Antimicrobial susceptibility profiles among the molecular types of MRSA isolates from pediatric patients**.

**Molecular type**	**Isolates^a^ (n)**	**CFZ^a^ (%)**	**LZD^a^ (%)**	**CIP^a^ (%)**	**DA^a^ (%)**	**E^a^ (%)**	**SXT^a^ (%)**	**MOF^a^ (%)**	**FD^a^ (%)**	**V^a^ (%)**	**TET^a^ (%)**	**P^a^ (%)**	**RD^a^ (%)**	**LEV^a^ (%)**	**AMP^a^ (%)**	**GM^a^ (%)**	**Q/D^a^ (%)**	**TGC^a^ (%)**
ST59	36	100	0	0	94.4	97.2	0	0	0	0	36.1	100	0	0	100	0	0	0
ST398	9	100	0	0	88.9	88.9	0	0	0	0	11.1	100	0	0	100	0	0	0
ST88	7	100	0	14.3	57.1	71.4	14.3	14.3	0	0	42.9	100	14.3	14.3	100	14.3	0	0
ST5	6	100	0	33.3	83.3	83.3	50	16.7	0	0	33.3	100	0	33.3	100	16.7	0	0
ST630	6	100	0	16.7	83.3	83.3	0	16.7	0	0	16.7	100	16.7	16.7	100	16.7	0	0
ST7	3	100	0	33.3	66.7	66.7	66.7	0	0	0	33.3	100	0	0	100	0	0	0
ST45	2	100	0	0	50	50	0	0	0	0	0	100	0	0	100	0	0	0
ST9	2	100	0	100	100	100	100	0	0	0	100	100	0	0	100	0	0	0
ST338	2	100	0	0	100	100	0	0	0	0	100	100	0	0	100	0	0	0
ST72	1	100	0	0	0	0	0	0	0	0	0	100	0	0	100	0	0	0
ST950	1	100	0	0	0	0	0	0	0	0	0	100	0	0	100	0	0	0
ST30	1	100	0	0	0	0	0	0	0	0	0	100	0	0	100	0	0	0
ST6	1	100	0	0	100	100	0	0	0	0	0	100	0	0	100	0	0	0
ST20	1	100	0	0	0	0	0	0	0	0	0	100	0	0	100	0	0	0
ST121	1	100	0	0	0	0	0	0	0	0	100	100	0	0	100	0	0	0
ST1507	1	100	0	0	0	0	0	0	0	0	0	100	0	0	100	0	0	0
Total	80	100	0	8.75	80	82.5	10	3.75	0	0	32.5	100	2.5	5	100	3.75	0	0

a *cefazolin (CFZ), linezolid (LZD), ciprofloxacin (CIP), clindamycin (DA), erythromycin (E), trimethoprim-sulfamethoxazole (SXT), moxifloxacin (MOF), nitrofurantoin (FD), vancomycin (V), tetracycline (TET), penicillin (P), rifampicin (RF), levofloxacin (LVX), ampicillin (AMP), gentamicin (GM), quinupristin/dalfopristin (Q/D) and tigecycline (TGC)*.

### Virulence gene profiles

The distribution of 19 putative virulence genes differed among the 80 MRSA strains according to MLST (Table 3). All virulence genes except *eta, etb*, and *arcA* genes were identified within multiple isolates, and all isolates exhibited carriage of at least 4 virulence genes. Thirty-four (42.5%, 34/80) isolates harbored ≥10 tested virulence genes, among which were 1 isolate with 13 genes, 3 isolates with 12 genes, 14 isolates with 11 genes, and 16 isolates with 10 genes.

**Table 3:** **Virulence gene distribution among the molecular types of MRSA isolates from pediatric patients**.

**Molecular type**	**Isolates (*n*)**	***icaA***	***clfA***	***sdrC***	***sdrE***	***hla***	***hlb***	***hlg***	***pvl***	***tsst***	***sea***	***seb***	***sec***	***sed***	***see***	***seg***	***seh***	***sei***	***sek***	***seq***
ST59	36	100	100	97.2	83.3	100	83.3	91.7	25	5.6	27.8	88.9	0	5.6	2.8	2.8	8.3	2.8	86.1	86.1
ST398	9	100	100	100	33.3	100	33.3	66.7	11.1	0	0	11.1	11.1	0	0	0	0	0	0	0
ST88	7	100	100	100	71.4	100	14.3	85.7	14.3	0	0	0	0	0	100	0	0	0	0	0
ST5	6	100	100	83.3	66.7	100	33.3	83.3	0	50	33.3	16.7	33.3	16.7	0	100	16.7	100	0	0
ST630	6	100	100	100	33.3	100	100	100	0	0	0	0	0	0	0	0	0	0	0	0
ST7	3	100	100	100	33.3	100	33.3	100	0	0	0	33.3	0	0	66.7	0	0	0	33.3	33.3
ST45	2	100	100	100	50	100	0	50	0	50	0	0	50	0	0	100	0	100	0	0
ST9	2	100	100	100	0	100	100	100	0	50	0	0	0	0	0	100	0	100	0	0
ST338	2	100	100	100	100	100	100	100	100	0	0	50	0	50	0	0	0	0	50	50
ST72	1	100	100	100	0	100	0	0	0	0	0	0	0	0	0	100	0	100	0	0
ST950	1	100	100	0	100	100	0	100	0	0	0	0	0	0	0	100	0	100	0	0
ST30	1	100	100	100	0	100	0	100	100	0	0	0	0	0	0	100	0	100	0	0
ST6	1	100	100	100	0	100	0	100	0	0	0	0	0	0	0	0	100	0	0	0
ST20	1	100	100	0	100	100	0	100	100	0	0	0	0	0	100	0	0	0	0	0
ST121	1	100	100	100	0	100	100	100	100	0	0	100	0	0	0	100	0	100	0	0
ST1507	1	100	100	100	100	100	0	100	0	0	0	0	0	0	0	100	0	100	0	0
Total	80	100	100	95	63.8	100	60	87.5	20	8.8	15	46.3	5	5	13.8	20	6.3	20	41.3	41.3

Adhesion genes were present in most of the MRSA isolates; 100% carried the *icaA* and *clfA* genes, 95% harbored *sdrC*, 63.8% carried *sdrE*. The most prevalent toxin genes detected were *hla* (100%), *hlg* (87.5%), *hlb* (60%), *seb* (46.3%), *sek* (41.3%), and *seq* (41.3%). The *pvl* gene was detected in 16 strains, which represented seven different STs, with ST59 being the most common.

### Molecular characteristics of the prevalent clone ST59

In this study, ST59 (45.0%, 36/80) was found to be the most prevalent clone, which was associated primarily with *spa t*437 (44.7%, 17/36) and *spa t*441 (18.4%, 7/36). ST59 strains were more resistant to erythromycin (*P* = 0.002) and clindamycin (*P* = 0.004) but more susceptible to ciprofloxacin (*P* = 0.015) and trimethoprim-sulfamethoxazole (*P* = 0.007) than other STs (Table 4). In addition, non-ST59 strains were more often associated with multiple antibiotic-resistance profiles. They were also resistant to moxifloxacin, rifampicin, levofloxacin, and gentamicin, while ST59 did not have these profiles.

**Table 4:** **Antimicrobial susceptibility profiles of ST59 and non-ST59 isolates**.

	**ST59 (*n* = 36), R^a^ (%)**	**Non-ST59 (*n* = 44), R (%)**	***P*- value^b^**
CFZ	100	100	
LZD	0	0	
CIP	0	15.9	<0.05
DA	94.4	68.1	<0.01
E	97.2	70.5	<0.01
SXT	0	18.2	<0.01
MOF	0	6.8	>0.05
FD	0	0	
V	0	0	
TET	36.1	29.5	>0.05
P	100	100	
RD	0	4.5	>0.05
LEV	0	9.1	>0.05
AMP	100	100	
GM	0	6.8	>0.05
Q/D	0	0	
TGC	0	0	

a *R, Resistance*.

b *The resistance rates of antimicrobials among ST59 were compared with those among non-ST59 isolates*.

All isolates exhibited *icaA, clfA*, and *hla* genes. The frequency of carriage for *hlb, sea, seb, sek, seq*, or *sdrE* among ST59 isolates was significantly higher than that for non-ST59 isolates (*P* < 0.05; Table 5). Twenty-eight (77.8%) of 36 ST59 isolates harbored ≥10 tested virulence genes, which was significantly higher than that among non-ST59 isolates (13.6%, 6/44) (*P* < 0.05). However, there were no significant differences on the positive rate of *pvl* between ST59 and non-ST59 strains.

**Table 5:** **Frequencies of virulence genes among ST59 and non-ST59 isolates**.

**Virulence genes**	**ST59 (*n* = 36)**	**Non-ST59 (*n* = 44)**	***P*-value^a^**
*icaA*	100	100	>0.05
*clfA*	100	100	>0.05
*sdrC*	97.2	93.2	>0.05
*sdrE*	83.3	47.7	<0.01
*hla*	100	100	>0.05
*hlb*	83.3	40.9	<0.01
*hlg*	91.7	84.1	>0.05
*pvl*	25	15.9	>0.05
*tsst*	5.6	11.4	>0.05
*sea*	27.8	4.5	<0.01
*seb*	88.9	11.4	<0.01
*sec*	0	9.1	
*sed*	5.6	4.5	>0.05
*see*	2.8	22.7	<0.01
*seg*	2.8	34.1	<0.01
*seh*	8.3	4.5	>0.05
*sei*	2.8	34.1	<0.01
*sek*	86.1	4.5	<0.01
*seq*	86.1	4.5	<0.01

a *The positive rates of virulence genes among ST59 were compared with those among non-ST59 isolates*.

## Discussion

Recently, CA-MRSA infection and transmission have become major public health problems worldwide, particularly among children and immunocompromised patients. Given the dangerous consequences of MRSA infection on pediatric patients, it is necessary to understand the prevalence, molecular characteristics and virulence profiles of these isolates in order to take effective measures to control infection and transmission in the affected communities.

Our data suggested that ST59-MRSA-IV and ST59-MRSA-V with *t*437 were still the most predominant clones among all of the clinical MRSA isolates in Shanghai. During recent years, CA-MRSA infections have been found worldwide and the major pandemic clones are usually related to specific geographical locations (Mediavilla et al., 2012; Li et al., 2016). For instance, the ST1 and ST8 clone in the USA and Canada, the ST30 clone in Australia, the ST80 clone in Europe, the ST59 clone in the Asia-Pacific region, including Taiwan and Australia. These five clones comprise most of the CA-MRSA worldwide. In China, previous studies of the most common genotypes revealed that ST59-MRSA-IV (and its single locus variant ST338-MRSA-IV) was the major lineage accounting for up to two-thirds of isolates and the most common *spa* type was *t*437 (Geng et al., 2010). Similar to these findings, the predominant types of MRSA isolates in our study were ST59-MRSA-IV and ST59-MRSA-V with *t*437. The ST59 MRSA clone was also prevalent in Taiwan, Hong Kong, Vietnam, Japan, and Australia (Chuang and Huang, 2013). Based on these finding, ST59 has become a successful clone with potential for clonal expansion. However, the CA-MRSA ST59 clone identified in mainland China might differ from that identified in Taiwan (Chen and Huang, 2005). Most PVL-positive CA-MRSA ST59 clones in Taiwan belong to SCC*mec* V_T_, while CA-MRSA ST59 clones in our study belong to SCC*mec* IV or SCC*mec* V. This observation supports the notion that ST59-MSSA lineages may provide a stable genetic environment for integration of SCC*mec* in favor of their infection and transmission in the community. In addition, the most prevalent multiresistant antibiotic profiles of ST59 strains included erythromycin, tetracycline, clindamycin, penicillin, and ampicillin. These findings may be explained by the selective pressure of antibiotic abuse in the Chinese community (Aires de Sousa et al., 2003).

In this study, ST398-MRSA- IV/V with three *spa* types, *t*034, *t*011, and *t*1250, was found to be the second most predominant type. This MRSA clone, first observed among pig and pig farmers in the Netherlands in 2003, then found in Austria, Germany, and Denmark (Fluit, 2012). Due to the presence of a restriction/methylation system, the strains are non-typeable with standard PFGE using SmaI digestion. ST398 is a typical livestock-associated type (Graveland et al., 2011), previous studies show that patients carrying this type are usually in contact with a major animal reservoir of these MRSA, mostly pigs (Yan et al., 2014). In our study, six patients (66.7%, 6/9) were <6 months and the others (33.3%, 3/9) were <3 years. From their medical history, they were no directly in contact with animal hosts, suggesting that there is a potential risk of MRSA introduction from the animal reservoirs into communities or hospitals with humans as the vector. ST9 is the dominant livestock-associated lineage in Asia (Chuang and Huang, 2015), there were two ST9 strains found in our study and no evidence to support them as LA-MRSA clones. In China, there is still very limited information on the occurrence of LA-MRSA infection, probably due to lack of diagnostic facilities and transmission information. Identifying risk factors and transmission routes is very important.

Based on available studies, the pathogenicity for *S. aureus* largely depends on the carriage of a battery of virulence factors associated with adhesion, invasion, persistence, the evasion/destruction of host defenses, tissue invasion/penetration and toxin-mediated disease (Moreillon et al., 1995; Bubeck Wardenburg et al., 2007; Diep and Otto, 2008; Yu et al., 2011). Of those, previous studies reported some virulence genes are found virtually in all *S. aureus*, while others are linked to specific molecular types. However, in our study, most of the MRSA isolates were found to harbor the *hla, hlg, seb*, and *sek* genes, but no association was found between molecular types and toxin genes. Compared to other STs, ST59 clones harbor more toxin genes and 77.8% of ST59 isolates harbored ≥10 tested virulence genes, which may help ST59 become successfully spread in China. In addition, the adhesion genes (*clfA, icaA, sdrC*, and *sdrE*) were evaluated in this study (Josefsson et al., 1998). All strains carried the *icaA* and *clfA* genes, 95% harbored *sdrC*, and 63.8% carried *sdrE*. The high percentage is consistent with the fact that these genes are ubiquitously carried by MRSA strains that belong to different lineages and that these genes have been reported to play a key role in the process of bacterial pathogenicity.

In conclusion, simultaneous carriage of multiple virulence genes and considerable genetic diversity were very common among CA-MRSA from pediatric patients in Shanghai. ST59-MRSA-IV with *t*437 was still the most predominant type. In an era of increasing CA-MRSA, appropriate measures need to be taken to prevent further morbidity and mortality worldwide. This effort should take into account the molecular characteristics of CA-MRSA strains.

## Author contributions

ML, QF, and XW designed the studies and obtained funding. XW, XL, WH, and WL performed the experiments and/or analyzed the data; ML, XW, and XL wrote the manuscript.

## Funding

This study was supported by the National Natural Science Foundation of China (grants 81301392) and the Training Program for Outstanding Young Teachers in Higher Education Institutions (ZZjdyx13132), the Training Program for Clinical Medical Young Talents in Shanghai, Visiting Scholar Research Program and SCMC-EPT Program to XW.

### Conflict of interest statement

The authors declare that the research was conducted in the absence of any commercial or financial relationships that could be construed as a potential conflict of interest.
